# Null genotypes of glutathione S-transferase μ1 and glutathione S-transferase θ1 are associated with osteosarcoma risk: A meta-analysis

**DOI:** 10.3892/ol.2015.2955

**Published:** 2015-02-10

**Authors:** JICHENG HAN, WEI DENG, LAIYING WANG, WANLI QI

**Affiliations:** Department of Orthopaedics, The Affiliated Hospital to Changchun University of Chinese Medicine, Changchun, Jilin 130000, P.R. China

**Keywords:** glutathione S-transferase, osteosarcoma, gene polymorphism

## Abstract

Glutathione S-transferase (GST) genetic polymorphisms has been reported to be associated with osteosarcoma; however, the results of previous studies are conflicting. Thus, in the present study, a meta-analysis was conducted to investigate the effects of GSTM1 and GSTT1 polymorphisms on osteosarcoma risk. A literature search was performed in the PubMed, Cochrane Library and China National Knowledge Infrastructure databases to identify case-control studies published prior to March 2014. Data were extracted and pooled odds ratios (OR) with 95% confidence intervals (CI) were calculated. In addition, Begg’s test was used to measure publication bias. Sensitivity analysis were performed to ensure the accuracy of the results. The meta-analysis results demonstrated no significant association between the null genotype of GSTM1 and osteosarcoma risk (OR=0.83; 95% CI, 0.37–1.85). By contrast, the results revealed a significant association for the comparison of null vs. non-null genotypes of GSTT1 (OR=1.54; 95% CI, 1.09–2.19). In conclusion, the GSTT1 null genotype may be associated with an increased risk of developing osteosarcoma. Further studies with larger sample sizes and well-designed methodologies are required to verify these conclusions.

## Introduction

Osteosarcoma is a highly malignant and aggressive bone tumor, occurring primarily in individuals between 10 and 30 years old (1). This tumor accounts for ~45% of all the bone sarcomas. Although five-year survival rates of 50–70% can be achieved through multimodal therapy, the lack of effective treatment options results in poor prognosis in a large number of patients (2). Further investigation of the pathogenesis of osteosarcoma is required to reduce the morbidity and mortality rates. A previous study has demonstrated that the established risk factors involved in the development of osteosarcoma include the following: Adolescent and young adult age, male gender, previous treatment with radiotherapy or anticancer drugs, particularly alkylating agents, and a family history of osteosarcoma (1). However, the etiology of osteosarcoma is not fully understood based on these risk factors; therefore, additional risk factors may be involved (3). Molecular biology studies have revealed strong evidence that genetic factors are important in the pathogenesis of osteosarcoma (4,5).

Glutathione S-transferases (GSTs) are a family of phase II enzymes (6). GSTs are mainly responsible for the detoxification of a wide range of environmental and nonenvironmental carcinogens (including polyaromatic hydrocarbons from second-hand cigarette smoke), chemotherapy agents (including alkylating agents and anthracyclines), inflammation-associated reactive oxygen species and metabolism-derived lipid peroxides (7). In addition, GSTs are able to modulate the induction of other enzymes and proteins that are important for cellular functions (8). Thus, GSTs are involved in the protection against various types of cellular damage and their malfunction may result in carcinogenesis.

In humans, the GST super family consists of numerous cytosolic, mitochondrial and microsomal proteins. Cytosolic proteins are divided into eight distinct classes, including the α, κ, μ, ω, π, σ, θ and ζ (9). The θ class of GSTs is encoded by the GST θ 1 (GSTT1) gene, which is located on chromosome 1p13.3 and contains 10 exons (10). In addition, the μ class is encoded by the GST μ1 (GSTM1) gene on chromosome 22q11.23 and contains six exons (11). Homozygous deletion (null genotype) is the most common variant of the GSTM1 and GSTT1 genes. In the Caucasian European populations, the prevalence of the GSTM1 deletion genotype is 47–58%, while the prevalence of the GSTT1 null genotype is 13–25% (12). A previous study has indicated that the null genotype may be associated with the absence of enzyme activity, increasing vulnerability to cytogenetic damage and resulting in susceptibility to cancer (13).

A previous study revealed that the null genotypes of GSTT1 and GSTM1 were associated with increased risk of bladder and prostate cancer (14). In addition, a number of studies have investigated the association between GSTM1 and GSTT1 polymorphisms and the risk of developing osteosarcoma (15,17,18). However, the results of these studies were inconsistent. A meta-analysis can be useful in the detection of an association that may not be identified in sample size studies, particularly studies evaluating rare allele frequency polymorphisms. The aim of the present study was to investigate the association between the null genotypes of GSTM1 and GSTT1 and the development of osteosarcoma by conducting a meta-analysis investigation of all the eligible case-control studies published to date.

## Materials and methods

### Literature search

The literature was screened (titles, abstracts and full text) by two researchers independently, in order to determine which studies were eligible for inclusion in this meta-analysis. The results were compared and disagreements were resolved by consensus. The PubMed (www.ncbi.nlm.nih.gov/pubmed), Cochrane Library (www.thecochranelibrary.com) and China National Knowledge Infrastructure (http://www.cnki.net) databases were examined to identify all the studies investigating the association between GSTM1 and GSTT1 polymorphisms and osteosarcoma risk, which were published prior to March 2014. The following key words were used: ‘glutathione S-transferases’, ‘GST’, ‘osteosarcoma’, ‘polymorphism’, ‘mutation’ and ‘variant’. No publication language restrictions were imposed. The references of all the studies identified through the literature search were investigated for other relevant publications. In the case that sequential or multiple publications using the same data were identified, the publication reporting data from the largest or most recent study was included. The search strategy used in this study is shown in Table I.

### Inclusion and exclusion criteria

The inclusion criteria for human studies included the following: i) Case-control studies, addressing osteosarcoma cases and healthy controls; ii) studies evaluating the association between GSTM1 and GSTT1 polymorphisms and osteosarcoma risk; and iii) studies that included sufficient genotype data for extraction. The exclusion criteria included the following: i) Non-case-control studies, evaluating the association between GSTM1 and GSTT1 polymorphisms and osteosarcoma risk; ii) case reports, letters, reviews, meta-analyses and editorial articles; iii) studies reporting incomplete or insufficient data; iv) studies containing duplicate data; and v) studies with a family-based design.

### Data extraction

The data were independently examined and extracted by two researchers, based on the aforementioned inclusion and exclusion criteria. In the case of inconsistency between the studies selected by the two researchers, consensus was reached following discussion. The following information was collected from the eligible studies: First author’s name; year of publication; country and ethnicity of the studied population; number of cases and controls; and number of genotyped cases and controls. The cases and controls were categorized into the Asian or Caucasian ethnic groups. No minimum number of patients was required to include a study in this meta-analysis.

### Statistical analyses

Odds ratios (ORs) with 95% confidence intervals (CIs) were used to determine the strength of association between the GSTM1 and GSTT1 polymorphisms and osteosarcoma risk. Pooled ORs for the risk associated with the GSTM1 and GSTT1 null genotypes vs. the non-null genotypes were calculated. Heterogeneities between the studies were estimated using the *I*^2^ test. *I*^2^ values of 25, 50 and 75% were defined as low, moderate and high estimates, respectively (15). When the *I*^2^ value was >50%, indicating heterogeneity across the studies, the random effects model was used for meta-analysis; otherwise, the fixed effects model was used. In addition, subgroup analysis based on ethnicity was used to investigate and interpret the diversity among the results of different studies. Sensitivity analysis was performed by using random effect values compared with the fixed effect in order to ensure the stability of the findings (16). Publication bias was investigated using Begg’s funnel plot and P<0.05 was considered to indicate a statistically significant publication bias. All the analyses were performed using the STATA version 12.0 software (StataCorp LP, College Station, TX, USA) and the significance level was set to 0.05.

## Results

### Identification of eligible studies

**Figure 1:**
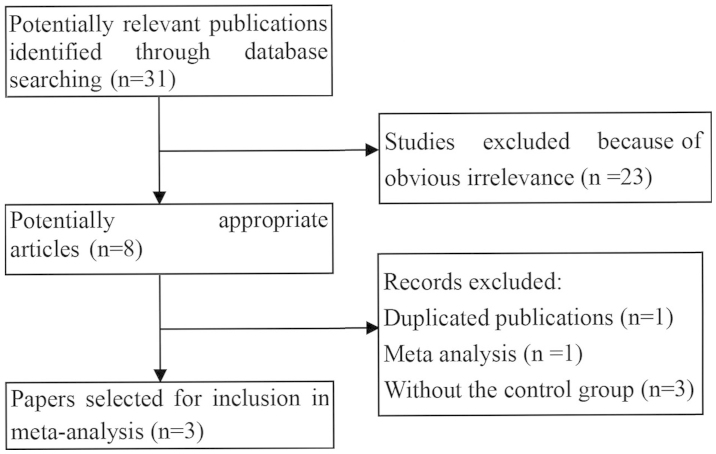
Flow chart of study study selection based on the inclusion and exclusion criteria.

Based on the search criteria used in the present study, 31 individual manuscripts were identified. Of these, eight full-text publications were preliminarily selected for further detailed evaluation. According to the exclusion criteria, five of these publications were then excluded, including one duplicate study, one meta-analysis and three studies with insufficient data for extraction. Finally, as shown in Fig. 1, three studies with 202 cases and 712 healthy controls were included in the current meta-analysis (17–19). A flow chart demonstrating the study selection process is summarized in Fig. 1. All the eligible studies were case-control studies that evaluated the association of the GSTM1 and GSTT1 null genotypes with the susceptibility to osteosarcoma. The publication year range of the included studies was between 2000 and 2014. The main characteristics of the eligible studies are summarized in Table I.

### Meta-analysis

**Figure 2:**
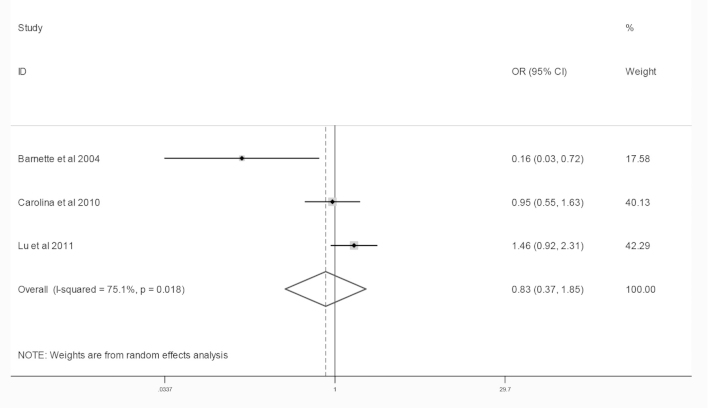
Meta-analysis of the association between the null genotype of glutathione S-transferase μ1 and osteosarcoma risk.

**Figure 3:**
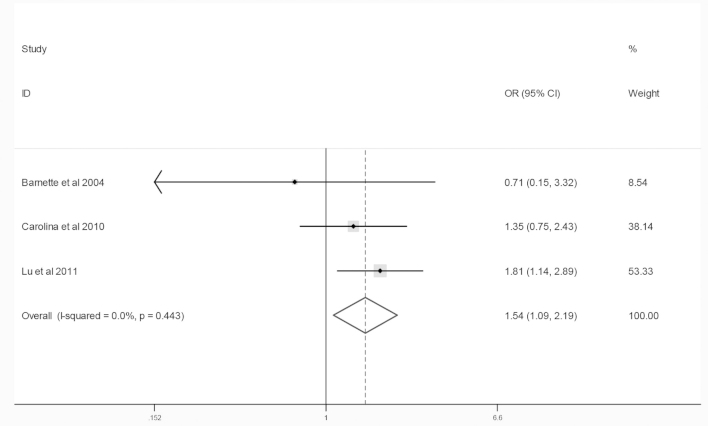
Meta-analysis of the association between the null genotype of glutathione S-transferase θ1 and osteosarcoma risk.

The combined results of the GSTM1 and GSTT1 null genotypes and osteosarcoma risk are summarized in Figs. 2 and 3 and Table II. The results of the meta-analysis revealed no association between the null genotypes of GSTM1 and the risk of osteosarcoma (OR=0.83; 95% CI, 0.37–1.85). By contrast, the meta-analysis indicated that the GSTT1 null genotype was associated with an increased risk of osteosarcoma in the two ethnic groups (OR=1.54; 95% CI, 1.09–2.19). Sensitivity analysis was performed by comparing the results of the fixed and random effects models. No alterations were detected in these results, indicating that the data of this meta-analysis were relatively stable and credible.

### Publication bias

The funnel plot and Begg’s test were used to assess the publication bias. No evidence of publication bias was detected in the present meta-analysis (P>0.05; Table II).

## Discussion

Even with identical environmental exposure, different individuals present a varied susceptibility to the same cancer type. A number of factors, including polymorphisms of genes involved in carcinogenesis, may account for this susceptibility variation. Therefore, recent studies have focused on genetic susceptibility to cancer (4,5). As important phase II enzymes, the GSTM1 and GSTT1 null genotypes are known to eliminate enzyme activities; therefore, these null genotypes have been linked with the increased number of cancer cases, possibly due to increased susceptibilities to environmental toxins and carcinogens (13).

The association between GSTM1 and GSTT1 null genotypes and osteosarcoma risk has been investigated in several studies (15,17,18); however, the results of these studies are controversial. The aim of meta-analyses is to combine similar studies in order to increase the sample size and statistical potential, obtaining more accurate results (20). To the best of our knowledge, the present study is the first systematic meta-analysis of the association between GSTM1 and GSTT1 null genotypes and osteosarcoma risk. The current study assessed quantitatively the association between the GSTM1 and GSTT1 null genotypes and susceptibility to osteosarcoma. In total, three case-control studies were found to be eligible and were investigated. These studies involved a total of 202 osteosarcoma cases and 712 healthy controls. The results revealed no statistically significant association between the GSTM1 null genotype and osteosarcoma risk (OR=0.83; 95%CI, 0.37–1.85). By contrast, the results demonstrated that the GSTT1 null genotype was significantly associated with the susceptibility to osteosarcoma (OR=1.54; 95% CI, 1.09–2.19). No evidence of publication bias was detected in this meta-analysis for the GSTM1 and GSTT1 null genotypes (P>0.05).

The underlying mechanism of the association between the GSTT1 null genotype and osteosarcoma risk remains unclear. A recent study demonstrated that GSTT1 is different compared with other phase II enzymes (such as GSTM1), which also exhibit phase I enzyme activity and possess the ability to activate carcinogens (17). The null genotype of GSTT1 is associated with the absence of enzyme activity and an increased cancer incidence. As the number of eligible studies selected in the present meta-analysis was small, these results require further verification.

The limitations of the current meta-analysis should be acknowledged. Firstly, the systematic review was based on unadjusted data, since the genotype information stratified for the main confounding variables was not available in the original studies, while the confounding factors addressed across the different studies were variable. In addition, this meta-analysis was not able to address all the sources of heterogeneity that existed among the previous studies for the majority of polymorphisms, although subgroup stratification analysis may be possible for the limited number of the included published studies. Furthermore, gene-gene and gene-environment interactions were not investigated in the present study, due to the lack of information from the original studies.

In conclusion, the present meta-analysis indicated that the GSTT1 null genotype was associated with an increased risk of developing osteosarcoma. Since only a small number of studies are available in this field and the current evidence remains limited, future studies with large sample groups and adequate methodological quality are required to obtain accurate results.

## Figures and Tables

**Table I:** Characteristics of literatures included in the meta-analysis.

Study included	Year	Country	Ethnicity	Genotyping	Cases/controls	Cases		Controls	

						Null	Non-null	Null	Non-null
GSTM1									
Barnette *et al*	2004	America	Caucasian	High-throughout assay	12/326	2	10	183	143
Carolina *et al*	2010	Brazil	Caucasian	PCR-RFLP	80/160	35	45	72	88
Lu *et al*	2011	China	Asian	TaqMan assay	110/226	61	49	104	122
GSTT1									
Barnette *et al*	2004	America	Caucasian	High-throughout assay	12/300	2	10	66	234
Carolina *et al*	2010	Brazil	Caucasian	PCR-RFLP	80/160	26	54	42	118
Lu *et al*	2011	China	Asian	TaqMan assay	110/226	70	40	111	115

GSTM1, glutathione S-transferase (GST) μ1; GSTT1, GST θ1; PCR, polymerase chain reaction; RFLP, restriction fragment length polymorphism.

**Table II:** Summary ORs and 95% CI of null genotypes of GSTM1 and GSTT1 and osteosarcoma risk.

Subgroup	Contrast	Type of effects model	Heterogeneity		Association		Publication bias	
		
			*I*^2^ (%)	P	OR	95% CI	z	P
GSTM1								
Total	Null vs. non-null	Random	75.1	0.02	0.83	0.37–1.85	0.00	1.00
GSTT1								
Total	Null vs. non-null	Fixed	0.0	0.44	1.54	1.09–2.19	0.00	1.00

GSTM1, glutathione S-transferase (GST) μ1; GSTT1, GST θ1.
